# Crystal structure of β-d,l-fructose

**DOI:** 10.1107/S2056989015016503

**Published:** 2015-09-12

**Authors:** Tomohiko Ishii, Tatsuya Senoo, Akihide Yoshihara, Kazuhiro Fukada, Genta Sakane

**Affiliations:** a Department of Advanced Materials Science, Faculty of Engineering, Kagawa University, 2217-20 Hayashi-cho, Takamatsu, Kagawa 761-0396, Japan; b Rare Sugar Research Center, Kagawa University, 2393 Ikenobe, Kagawa 761-0795, Japan; c Department of Applied Biological Science, Faculty of Agriculture, Kagawa University, 2393 Ikenobe, Kagawa 761-0795, Japan; d Department of Chemistry, Faculty of Science, Okayama University of Science, 1-1 Ridaicho, Kita-ku, Okayama 700-0005, Japan

**Keywords:** crystal structure, hydrogen bonding, racemic compound, rare sugar

## Abstract

The title compound, C_6_H_12_O_6_, was crystallized from an aqueous solution of equimolar mixture of d- and l-fructose (1,3,4,5,6-penta­hydroxy­hexan-2-one, *arabino*-hexulose or levu­lose), and it was confirmed that d-fructose (or l-fructose) formed β-pyran­ose with a ^2^ *C*_5_ (or ^5^ *C*_2_) conformation. In the crystal, two O—H⋯O hydrogen bonds between the hy­droxy groups at the C-1 and C-3 positions, and at the C-4 and C-5 positions connect homochiral mol­ecules into a column along the *a* axis. The columns are linked by other O—H⋯O hydrogen bonds between d- and l-fructose mol­ecules, forming a three-dimensional network.

## Related literature   

For crystal structures of chiral β-d-fructose, racemic β-d,l-allose and racemic β-d,l-psicose, see: Kanters *et al.* (1977 ▸); Ishii, Senoo *et al.* (2015 ▸); Ishii, Sakane *et al.* (2015 ▸), respectively. For the synthesis of chiral l-fructose, see: Itoh & Izumori (1996 ▸).
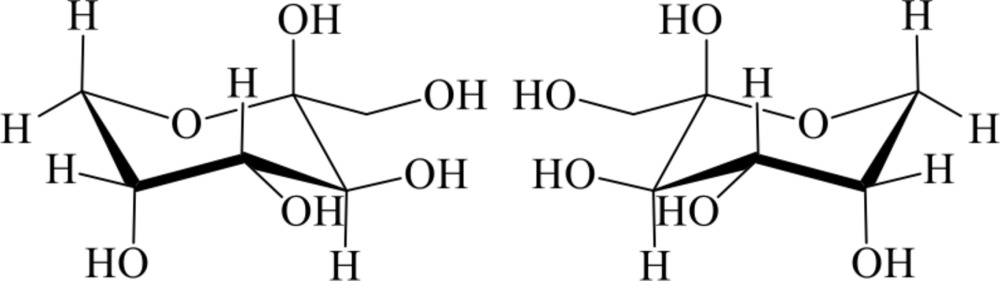


## Experimental   

### Crystal data  

C_6_H_12_O_6_
*M* *_r_* = 180.16
Triclinic, 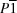

*a* = 5.43124 (19) Å
*b* = 7.2727 (3) Å
*c* = 10.1342 (4) Å
α = 69.120 (2)°
β = 83.907 (2)°
γ = 78.381 (2)°
*V* = 366.09 (2) Å^3^
*Z* = 2
Cu *K*α radiation
μ = 1.30 mm^−1^
*T* = 296 K
0.10 × 0.10 × 0.10 mm

### Data collection  

Rigaku R-AXIS RAPID diffractometer
Absorption correction: multi-scan (*ABSCOR*; Rigaku, 1995 ▸) *T*_min_ = 0.729, *T*_max_ = 0.878
6710 measured reflections
1329 independent reflections
1211 reflections with *F* ^2^ > 2.0σ(*F* ^2^)
*R*_int_ = 0.079

### Refinement  

*R*[*F* ^2^ > 2σ(*F* ^2^)] = 0.037
*wR*(*F* ^2^) = 0.095
*S* = 1.08
1329 reflections
115 parameters
H-atom parameters constrained
Δρ_max_ = 0.32 e Å^−3^
Δρ_min_ = −0.23 e Å^−3^

### 

Data collection: *RAPID-AUTO* (Rigaku, 2009 ▸); cell refinement: *RAPID-AUTO*; data reduction: *RAPID-AUTO*; program(s) used to solve structure: *SIR2011* (Burla *et al.*, 2012 ▸); program(s) used to refine structure: *SHELXL2013* (Sheldrick, 2015 ▸); molecular graphics: *CrystalStructure* (Rigaku, 2014 ▸); software used to prepare material for publication: *CrystalStructure*.

## Supplementary Material

Crystal structure: contains datablock(s) global, I. DOI: 10.1107/S2056989015016503/is5416sup1.cif

Structure factors: contains datablock(s) I. DOI: 10.1107/S2056989015016503/is5416Isup2.hkl

Supporting information file. DOI: 10.1107/S2056989015016503/is5416Isup3.cml

**Fig. 1.**
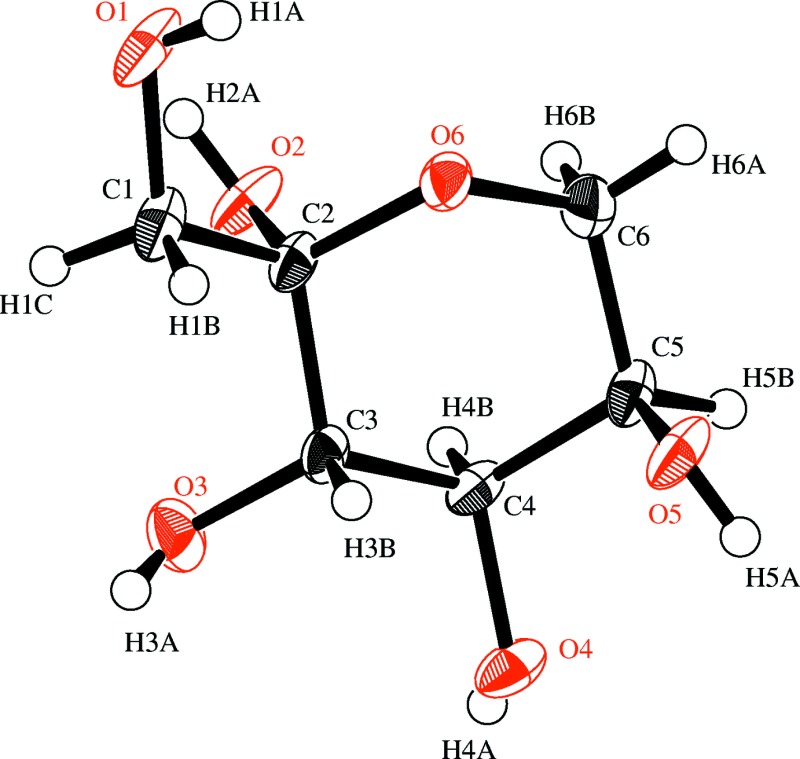
ORTEP view of the title compound with the atom-labeling scheme. The thermal ellipsoids of all non-hydrogen atoms are drawn at the 50% probability level. H atoms are shown as small spheres of arbitrary radius.

**Fig. 2.**
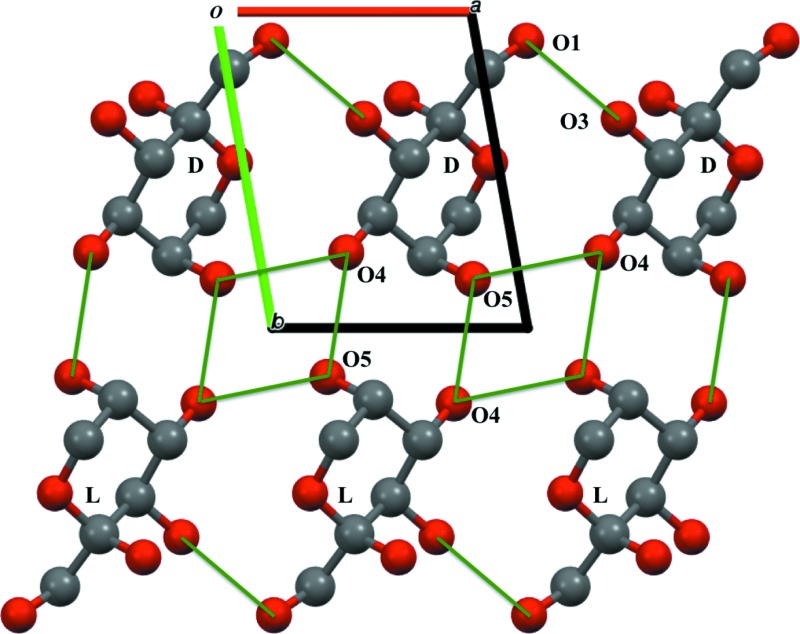
Part of the packing diagram of the title compound viewed down the c-axis, showing the hydrogen-bonding network (green solid lines).

**Fig. 3.**
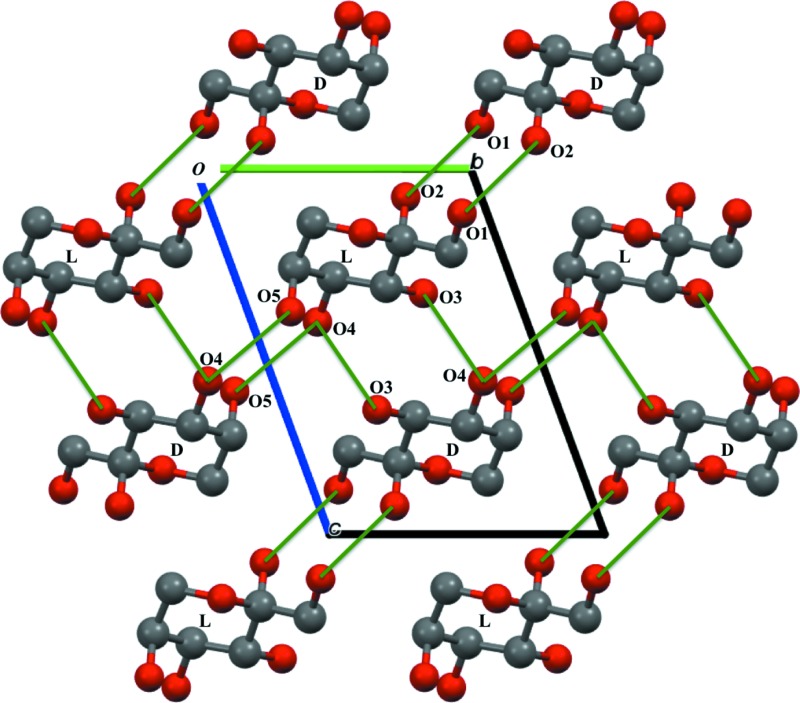
Part of the packing diagram of the title compound viewed down the a-axis, showing the hydrogen-bonding network (green solid lines).

ORTEP . DOI: 10.1107/S2056989015016503/is5416fig1.tif
*ORTEP* view of the title compound with the atom-labeling scheme. The thermal ellipsoids of all non-hydrogen atoms are drawn at the 50% probability level. H atoms are shown as small spheres of arbitrary radius.

c . DOI: 10.1107/S2056989015016503/is5416fig2.tif
Part of the packing diagram of the title compound viewed down the *c*-axis, showing the hydrogen-bonding network (green solid lines).

a . DOI: 10.1107/S2056989015016503/is5416fig3.tif
Part of the packing diagram of the title compound viewed down the *a*-axis, showing the hydrogen-bonding network (green solid lines).

CCDC reference: 1422317

Additional supporting information:  crystallographic information; 3D view; checkCIF report

## Figures and Tables

**Table 1:** Hydrogen-bond geometry (, )

*D*H*A*	*D*H	H*A*	*D* *A*	*D*H*A*
O1H1*A*O3^i^	0.82	2.28	2.9202(14)	135
O2H2*A*O1^ii^	0.82	1.93	2.7224(13)	161
O3H3*A*O4^iii^	0.82	1.96	2.7831(18)	177
O4H4*A*O5^iv^	0.82	2.01	2.7893(13)	158
O5H5*A*O4^v^	0.82	2.05	2.8431(12)	163

Symmetry codes: (i) 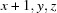; (ii) 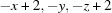; (iii) 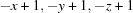; (iv) 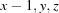; (v) 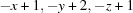.

## supplementary crystallographic information

### S1. Comment

Fructose, especially in *D*-fructose, is one of the most famous, fundamental and important monosaccharides in sugar family, and has been under intense investigation. On the other hand, *L*-fructose is classified into a rare sugar, and hardly exists in nature. In this study we investigate to create a novel racemic single-crystal including these *D*- and *L*-fructoses together with the ratio of 1: 1. The space group is triclinic *P*1 (*Z* = 2), which is significantly different from our previous reports of the racemic β-*D,L*-allose (monoclinic *P*2_1_/*c*, *Z* = 4; Ishii, Senoo *et al.*, 2015) and psicose (orthorhombic *Pna*2_1_, *Z* = 4; Ishii, Sakane *et al.*, 2015). In the unit cell, the *D*- and *L*-molecules are located with the heterochiral hydrogen bonding networks (O3—H3A···O4). As shown in Fig. 2, two homochiral hydrogen bonding networks (O1—H1A···O3 and O4—H4A···O5) have also been observed along to the *a*-axis. Additional two heterochiral hydrogen bonds (O2—H2A···O1 and O5—H5A···O4) are also confirmed (Fig. 3).

### S2. Experimental

*D*-Fructose was purchased from Wako Pure Chemical Industries. *L*-Fructose was prepared from *L*-psicose by enzymatic epimerization using *D*-tagatose 3-epimerase (Itoh & Izumori, 1996). *D*-Fructose and *L*-fructose were mixed in equal amount and dissolved in hot water to give a 70 wt% solution. And these samples were kept at room temperature. After one day, small single crystals were obtained in a hermetically sealed test tube.

### S3. Refinement

H atoms bounded to methine-type C (H3B, H4B, H5B) and methylene-type C (H1B, H1C, H6A, H6B) were positioned geometrically with C—H = 0.98 and 0.97 Å, respectively, and refined using a riding model with *U*_iso_(H) = 1.2*U*_eq_(C). H atoms bounded to O (H1A, H2A, H3A, H4A, H5A) were positioned geometrically (O—H = 0.82 Å) and refined as riding with *U*_iso_(H) = 1.2*U*_eq_(O), allowing for free rotation of the OH groups.

### Crystal data

**Table:**

C_6_H_12_O_6_	*Z* = 2
*M**_r_* = 180.16	*F*(000) = 192.00
Triclinic, *P*1	*D*_x_ = 1.634 Mg m^−^^3^
*a* = 5.43124 (19) Å	Cu *K*α radiation, λ = 1.54187 Å
*b* = 7.2727 (3) Å	Cell parameters from 4534 reflections
*c* = 10.1342 (4) Å	θ = 4.7–68.4°
α = 69.120 (2)°	µ = 1.30 mm^−^^1^
β = 83.907 (2)°	*T* = 296 K
γ = 78.381 (2)°	Block, colorless
*V* = 366.09 (2) Å^3^	0.10 × 0.10 × 0.10 mm

### Data collection

**Table:**

Rigaku R-AXIS RAPID diffractometer	1211 reflections with *F*^2^ > 2.0σ(*F*^2^)
Detector resolution: 10.000 pixels mm^-1^	*R*_int_ = 0.079
ω scans	θ_max_ = 68.2°, θ_min_ = 4.7°
Absorption correction: multi-scan (*ABSCOR*; Rigaku, 1995)	*h* = −6→6
*T*_min_ = 0.729, *T*_max_ = 0.878	*k* = −8→8
6710 measured reflections	*l* = −12→12
1329 independent reflections	

### Refinement

**Table:**

Refinement on *F*^2^	Hydrogen site location: inferred from neighbouring sites
*R*[*F*^2^ > 2σ(*F*^2^)] = 0.037	H-atom parameters constrained
*wR*(*F*^2^) = 0.095	*w* = 1/[σ^2^(*F*_o_^2^) + (0.0374*P*)^2^ + 0.1404*P*] where *P* = (*F*_o_^2^ + 2*F*_c_^2^)/3
*S* = 1.08	(Δ/σ)_max_ < 0.001
1329 reflections	Δρ_max_ = 0.32 e Å^−^^3^
115 parameters	Δρ_min_ = −0.23 e Å^−^^3^
0 restraints	Extinction correction: *SHELXL2013* (Sheldrick, 2015)
Primary atom site location: structure-invariant direct methods	Extinction coefficient: 0.125 (6)
Secondary atom site location: difference Fourier map	

### Special details

**Table:**

Refinement. Refinement was performed using all reflections. The weighted *R*-factor (*wR*) and goodness of fit (*S*) are based on *F*^2^. *R*-factor (gt) are based on *F*. The threshold expression of *F*^2^ > 2.0 σ(*F*^2^) is used only for calculating *R*-factor (gt).

### Fractional atomic coordinates and isotropic or equivalent isotropic displacement parameters (Å^2^)

**Table:**

	*x*	*y*	*z*	*U*_iso_*/*U*_eq_	
O1	1.19555 (19)	0.09152 (15)	0.87814 (12)	0.0325 (3)	
O2	0.66671 (18)	0.27440 (15)	0.92243 (11)	0.0287 (3)	
O3	0.50386 (19)	0.33842 (16)	0.65941 (12)	0.0294 (3)	
O4	0.34223 (18)	0.76120 (15)	0.58108 (11)	0.0264 (3)	
O5	0.81908 (18)	0.84537 (14)	0.60893 (12)	0.0290 (3)	
O6	0.96847 (17)	0.47716 (14)	0.82413 (11)	0.0223 (3)	
C1	1.0136 (3)	0.1818 (2)	0.77318 (17)	0.0265 (4)	
C2	0.8236 (2)	0.34747 (19)	0.80373 (15)	0.0195 (3)	
C3	0.6557 (2)	0.46432 (19)	0.67791 (15)	0.0190 (3)	
C4	0.4906 (3)	0.6455 (2)	0.70256 (15)	0.0200 (3)	
C5	0.6558 (3)	0.77295 (19)	0.72899 (16)	0.0223 (3)	
C6	0.8182 (3)	0.6457 (2)	0.85210 (16)	0.0254 (4)	
H1A	1.30779	0.15812	0.86062	0.0390*	
H1C	0.92594	0.08085	0.76811	0.0318*	
H1B	1.0976	0.23653	0.68208	0.0318*	
H2A	0.74136	0.16995	0.97716	0.0345*	
H3A	0.55367	0.31042	0.58847	0.0353*	
H3B	0.76216	0.5097	0.59244	0.0227*	
H4A	0.19353	0.75392	0.60178	0.0317*	
H4B	0.37832	0.60041	0.78545	0.0240*	
H5A	0.74552	0.94902	0.55285	0.0348*	
H5B	0.55062	0.8858	0.75058	0.0268*	
H6A	0.92669	0.72507	0.86923	0.0305*	
H6B	0.71231	0.59981	0.93633	0.0305*	

### Atomic displacement parameters (Å^2^)

**Table:**

	*U*^11^	*U*^22^	*U*^33^	*U*^12^	*U*^13^	*U*^23^
O1	0.0202 (6)	0.0153 (5)	0.0465 (8)	−0.0003 (4)	−0.0014 (5)	0.0066 (5)
O2	0.0251 (6)	0.0190 (5)	0.0265 (6)	0.0006 (4)	0.0037 (5)	0.0074 (4)
O3	0.0292 (6)	0.0280 (6)	0.0361 (7)	−0.0153 (5)	−0.0002 (5)	−0.0118 (5)
O4	0.0160 (5)	0.0236 (6)	0.0298 (6)	−0.0001 (4)	−0.0051 (4)	0.0019 (5)
O5	0.0208 (5)	0.0154 (5)	0.0385 (7)	−0.0035 (4)	−0.0014 (5)	0.0060 (5)
O6	0.0186 (5)	0.0164 (5)	0.0309 (6)	−0.0004 (4)	−0.0061 (4)	−0.0071 (4)
C1	0.0223 (7)	0.0144 (7)	0.0389 (9)	−0.0022 (6)	−0.0001 (6)	−0.0054 (6)
C2	0.0176 (7)	0.0123 (6)	0.0242 (8)	−0.0039 (5)	0.0003 (6)	−0.0007 (5)
C3	0.0177 (7)	0.0140 (7)	0.0233 (8)	−0.0070 (5)	−0.0006 (6)	−0.0020 (6)
C4	0.0158 (7)	0.0163 (7)	0.0213 (8)	−0.0018 (5)	−0.0019 (6)	0.0013 (6)
C5	0.0198 (7)	0.0131 (7)	0.0315 (9)	−0.0005 (5)	−0.0002 (6)	−0.0060 (6)
C6	0.0272 (8)	0.0199 (7)	0.0304 (8)	−0.0015 (6)	−0.0044 (6)	−0.0107 (6)

### Geometric parameters (Å, º)

**Table:**

O1—C1	1.4153 (19)	O1—H1A	0.820
O2—C2	1.4017 (16)	O2—H2A	0.820
O3—C3	1.419 (2)	O3—H3A	0.820
O4—C4	1.4395 (16)	O4—H4A	0.820
O5—C5	1.4305 (17)	O5—H5A	0.820
O6—C2	1.423 (2)	C1—H1C	0.970
O6—C6	1.4298 (19)	C1—H1B	0.970
C1—C2	1.520 (2)	C3—H3B	0.980
C2—C3	1.5304 (19)	C4—H4B	0.980
C3—C4	1.521 (2)	C5—H5B	0.980
C4—C5	1.517 (2)	C6—H6A	0.970
C5—C6	1.5075 (19)	C6—H6B	0.970
			
C2—O6—C6	113.25 (10)	C4—O4—H4A	109.467
O1—C1—C2	111.97 (14)	C5—O5—H5A	109.473
O2—C2—O6	111.23 (13)	O1—C1—H1C	109.215
O2—C2—C1	112.44 (10)	O1—C1—H1B	109.220
O2—C2—C3	107.69 (10)	C2—C1—H1C	109.218
O6—C2—C1	105.55 (11)	C2—C1—H1B	109.221
O6—C2—C3	109.06 (10)	H1C—C1—H1B	107.907
C1—C2—C3	110.86 (13)	O3—C3—H3B	109.026
O3—C3—C2	109.62 (10)	C2—C3—H3B	109.028
O3—C3—C4	110.08 (11)	C4—C3—H3B	109.024
C2—C3—C4	110.04 (13)	O4—C4—H4B	109.255
O4—C4—C3	110.05 (13)	C3—C4—H4B	109.253
O4—C4—C5	109.74 (10)	C5—C4—H4B	109.254
C3—C4—C5	109.27 (11)	O5—C5—H5B	109.808
O5—C5—C4	110.97 (14)	C4—C5—H5B	109.810
O5—C5—C6	107.71 (11)	C6—C5—H5B	109.807
C4—C5—C6	108.69 (11)	O6—C6—H6A	109.512
O6—C6—C5	110.67 (14)	O6—C6—H6B	109.511
C1—O1—H1A	109.469	C5—C6—H6A	109.511
C2—O2—H2A	109.478	C5—C6—H6B	109.513
C3—O3—H3A	109.476	H6A—C6—H6B	108.082
			
C2—O6—C6—C5	−61.73 (13)	C1—C2—C3—C4	−172.62 (10)
C6—O6—C2—O2	−58.74 (12)	O3—C3—C4—O4	−62.10 (13)
C6—O6—C2—C1	179.03 (9)	O3—C3—C4—C5	177.33 (9)
C6—O6—C2—C3	59.88 (13)	C2—C3—C4—O4	176.97 (10)
O1—C1—C2—O2	−68.49 (15)	C2—C3—C4—C5	56.41 (12)
O1—C1—C2—O6	52.95 (13)	O4—C4—C5—O5	−58.98 (13)
O1—C1—C2—C3	170.90 (9)	O4—C4—C5—C6	−177.25 (10)
O2—C2—C3—O3	−57.21 (15)	C3—C4—C5—O5	61.77 (12)
O2—C2—C3—C4	63.99 (14)	C3—C4—C5—C6	−56.50 (14)
O6—C2—C3—O3	−178.02 (10)	O5—C5—C6—O6	−61.97 (15)
O6—C2—C3—C4	−56.82 (13)	C4—C5—C6—O6	58.34 (14)
C1—C2—C3—O3	66.18 (13)		

### Hydrogen-bond geometry (Å, º)

**Table:**

*D*—H···*A*	*D*—H	H···*A*	*D*···*A*	*D*—H···*A*
O1—H1*A*···O3^i^	0.82	2.28	2.9202 (14)	135
O2—H2*A*···O1	0.82	2.60	2.9721 (14)	110
O2—H2*A*···O1^ii^	0.82	1.93	2.7224 (13)	161
O3—H3*A*···O4^iii^	0.82	1.96	2.7831 (18)	177
O4—H4*A*···O5^iv^	0.82	2.01	2.7893 (13)	158
O5—H5*A*···O4^v^	0.82	2.05	2.8431 (12)	163

Symmetry codes: (i) *x*+1, *y*, *z*; (ii) −*x*+2, −*y*, −*z*+2; (iii) −*x*+1, −*y*+1, −*z*+1; (iv) *x*−1, *y*, *z*; (v) −*x*+1, −*y*+2, −*z*+1.
