# Supplementary figures and images for: Crystal structure of β-d,l-fructose
Source: Acta Crystallogr E Crystallogr Commun. 2015 Sep 12;71(Pt 10):o719–20. doi: 10.1107/S2056989015016503 (PMC4647344; doi:10.1107/S2056989015016503)

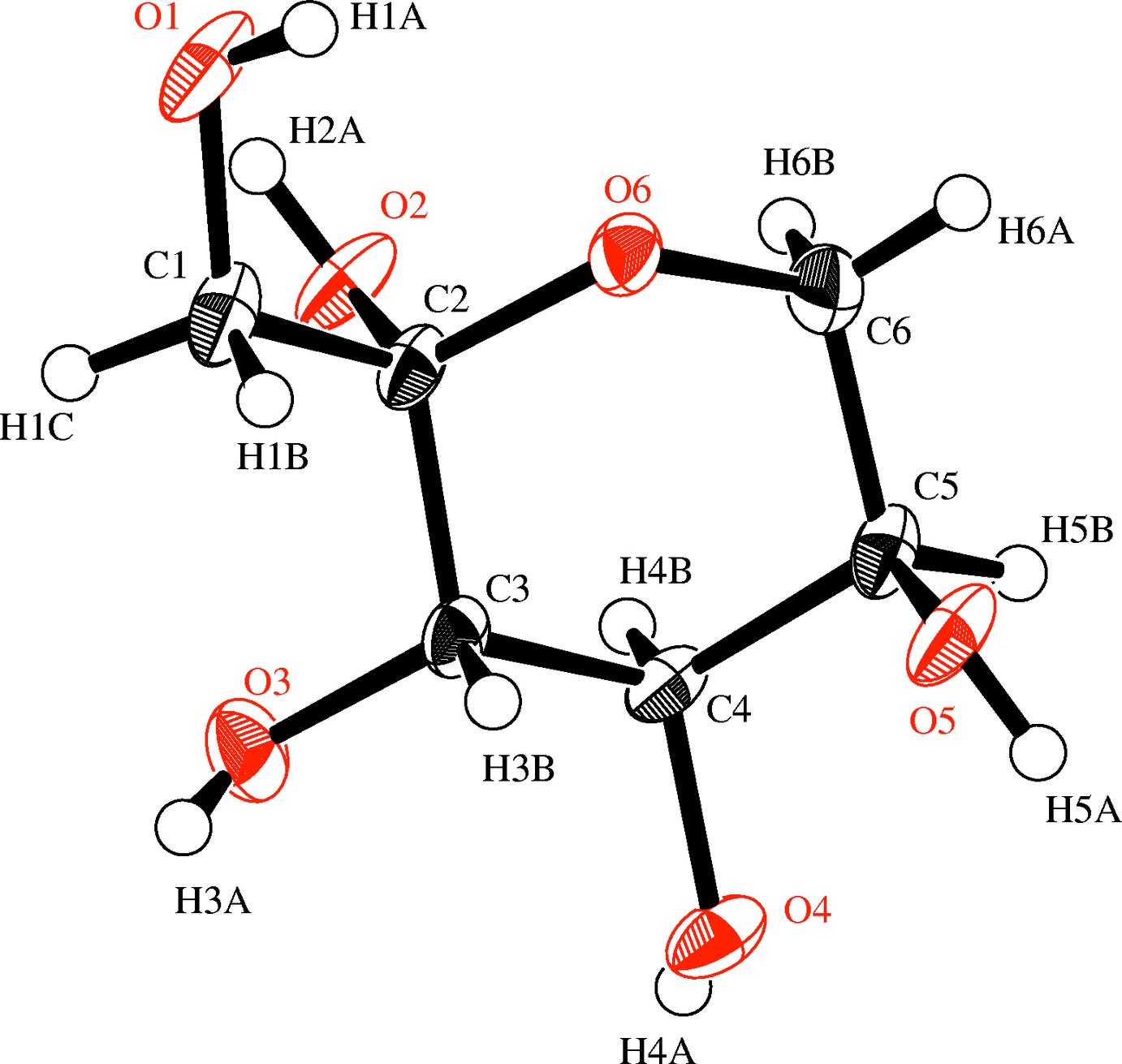

Supplement: Supplementary file 4 [file e-71-0o719-fig1.tif]

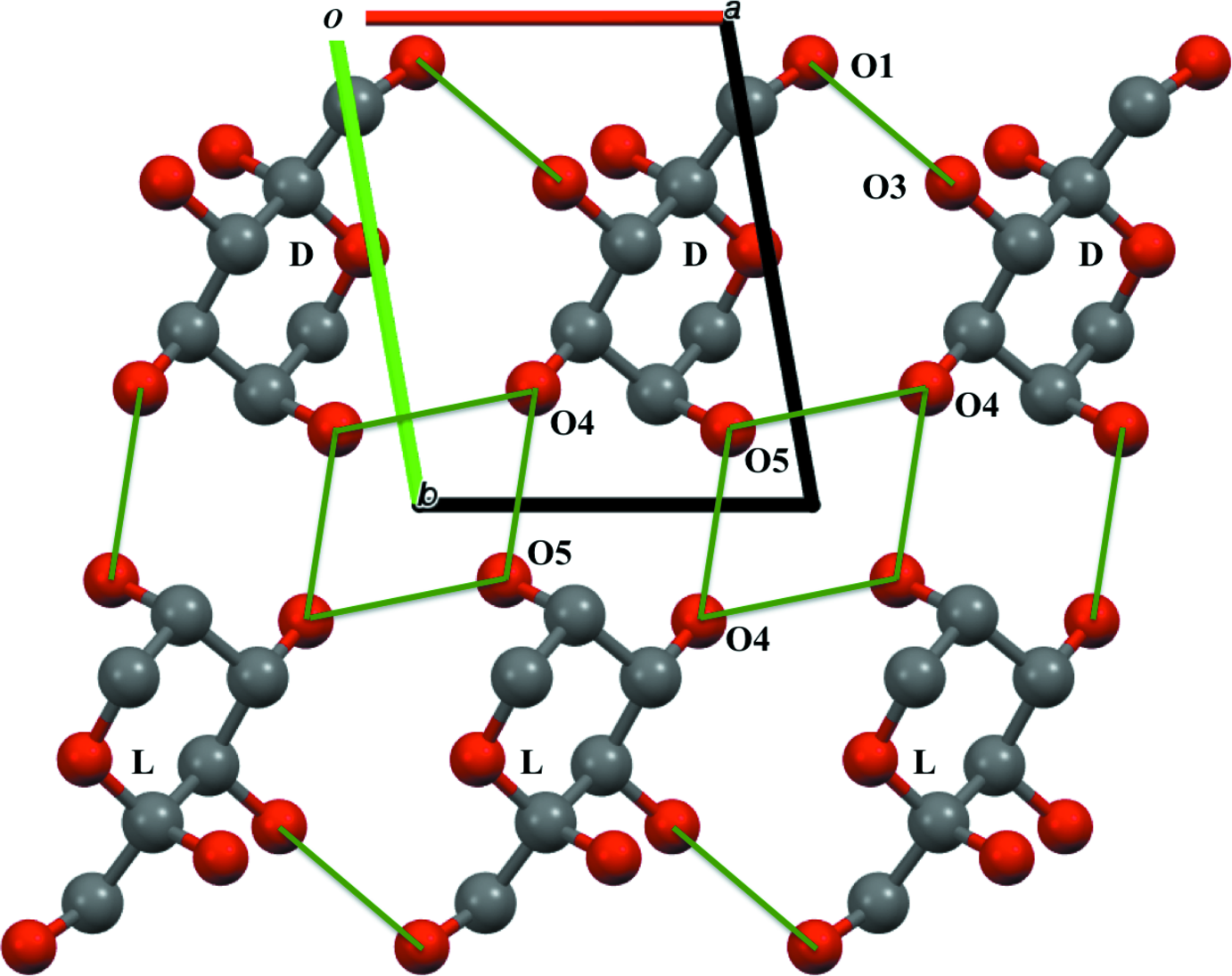

Supplement: Supplementary file 5 [file e-71-0o719-fig2.tif]

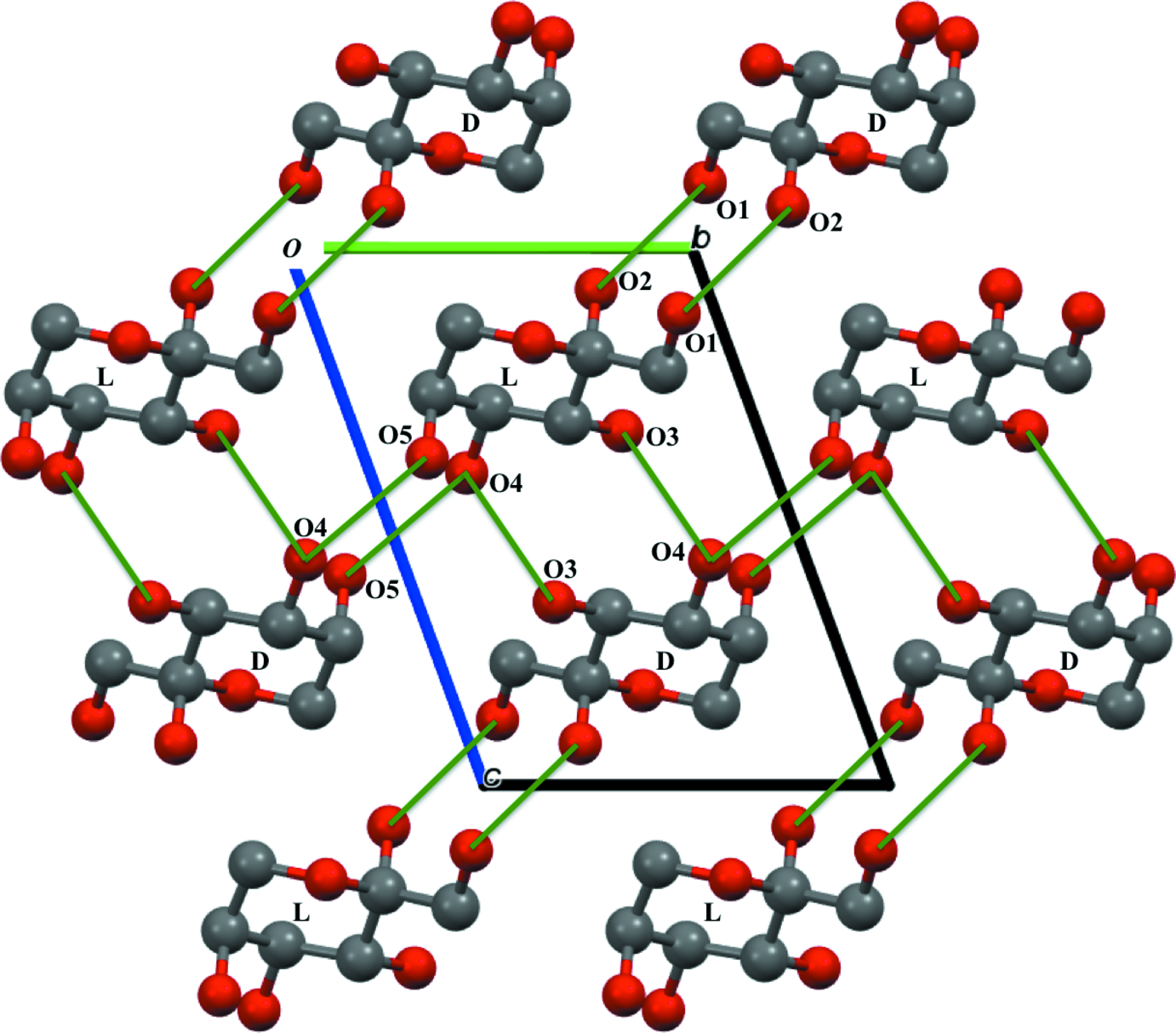

Supplement: Supplementary file 6 [file e-71-0o719-fig3.tif]
